# Effect of Fiber Type and Content on Mechanical Property and Lapping Machinability of Fiber-Reinforced Polyetheretherketone

**DOI:** 10.3390/polym14061079

**Published:** 2022-03-08

**Authors:** Shang Gao, Jialu Qu, Honggang Li, Renke Kang

**Affiliations:** Key Laboratory for Precision and Non-Traditional Machining Technology of Ministry of Education, Dalian University of Technology, Dalian 116024, China; gaoshang@dlut.edu.cn (S.G.); 21904049@mail.dlut.edu.cn (J.Q.); superhonggang@mail.dlut.edu.cn (H.L.)

**Keywords:** polyetheretherketone, short fiber-reinforced, material property, lapping machinability

## Abstract

Polyetheretherketone (PEEK) is a novel polymer material with excellent material properties. The hardness and strength of PEEK can be further improved by introducing fiber reinforcements to meet the high-performance index of the aerospace industry. The machinability will be influenced when the material properties change. Therefore, it is crucial to investigate the influence of material properties of the fiber-reinforced PEEK on machinability. In this paper, the main materials include pure PEEK, short carbon-fiber-reinforced PEEK (CF/PEEK), and short glass-fiber-reinforced PEEK (GF/PEEK). The effects of the fiber type and mass fraction on the tensile strength, hardness, and elastic modulus of materials were discussed using the tensile test and nanoindentation experiments. Furthermore, the fiber-reinforced PEEK lapping machinability was investigated using lapping experiments with abrasive papers of different mesh sizes. The results showed that the hardness and elastic modulus of PEEK could be improved with fiber mass fraction, and the tensile strength of CF/PEEK can be improved compared with that of GF/PEEK. In terms of lapping ability, the material removal rates of the fiber-reinforced materials were found to be lower than the pure PEEK due to the higher hardness of the fiber. During the lapping process, the material removal methods mainly included the ductile deformation or desquamation of reinforcing fiber and ductile removal of the PEEK matrix. The lapped surface roughness of PEEK material can be improved by fiber reinforcement.

## 1. Introduction

Polyetheretherketone (PEEK) is a novel crystalline thermoplastic polymer material that is widely used in aerospace, electronics, and medical industries for its excellent properties, such as low density and superior machinability [1,2,3]. However, PEEK has a few shortcomings, such as lower strength and lower hardness than most metal materials. These make it difficult to meet the higher performance requirements of certain industries. Therefore, the study about the performance of modified PEEK is of great significance. The mechanical properties of PEEK can be further improved through modification, and fiber-reinforced modification is a commonly used method to modify PEEK. The material properties of the fiber-reinforced PEEK vary with different fiber types. The fibers commonly used for PEEK reinforcement include carbon fiber, glass fiber, graphite fiber, and polytetrafluoroethylene (PTFE) fiber [4,5,6,7]. Carbon fiber has certain advantages, such as high strength, high modulus, small thermal expansion coefficient, and superior machinability [8,9]. On the other hand, glass fiber is often preferred due to its high stiffness, elastic modulus, and good load-bearing capacity [10]. Both carbon and glass fibers are widely used for reinforcement purposes [11,12,13]. However, despite materials possessing excellent properties, they also need to obtain better surface integrity through mechanical processing to fulfill the high requirements of the manufacturing industry [14]. Therefore, to expand the application fields of PEEK materials, it is crucial to study the material properties of the fiber-reinforced PEEK and analyze the influences of the change in the material properties on machinability.

The material properties and machinability of PEEK material change with fiber reinforcement, and the types and the mass fraction of fibers have diverse impacts on the PEEK matrix. The mechanical properties of PEEK can be enhanced with fiber reinforcing. Li et al. [9] compared the mechanical properties of PEEK and carbon-fiber-reinforced PEEK (CF/PEEK) and pointed out that carbon fiber can significantly improve the hardness, tensile, and compressive strength of PEEK. Zhang et al. [15] studied the material properties enhancement of the PEEK matrix by analyzing various fibers. The results showed that carbon fiber, glass fiber, and TiO_2_ could effectively increase the tensile strength of the PEEK matrix. Although the material properties of PEEK have been improved by fiber reinforcement, PEEK material still needs to be mechanically processed to meet the higher surface integrity requirements of some industries. Therefore, it is of great significance to study the machinability of the fiber-reinforced PEEK materials. The commonly applied machining methods of modified PEEK materials include turning and grinding. Davim et al. [14] studied the influence of turning parameters on cutting force and surface roughness of PEEK and glass-fiber–reinforced PEEK (GF/PEEK) materials with PCD tools turning. The results showed that the cutting force decreased with the increase in cutting velocity and feed rate. The surface roughness of PEEK and GF/PEEK decreased with increasing cutting velocity and increased with the feed rate. The surface quality of pure PEEK was better than GF/PEEK with the same turning parameters. Ji et al. [16,17] worked on the nanomechanical properties and machinability with the single-point diamond turning of PEEK, CF/PEEK, and GF/PEEK materials. The results demonstrated that PEEK was a single-phase material with constant values of nano-hardness and modulus, whereas CF/PEEK and GF/PEEK were fiber composite materials with superior hardness and elastic modulus. The processing surface had poor uniformity of force in turning, which led to poorer turning processability as compared to the pure PEEK. Khoran et al. [18] investigated the grinding machinability of PEEK materials. They concluded that the ground temperature has a great influence on grinding surface morphology and force. The ground surface quality was profoundly affected by the cryogenic cooling that led to superior surface quality.

Although micron surface roughness of PEEK materials can be achieved by turning and grinding, the surface roughness after processing was bad due to the poor uniformity of the cutting force. Therefore, ultra-precision lapping is suitable for PEEK material processing due to its good uniformity and controllability of the lapping force, and improved machinability is achieved on the surface of the workpiece. Furthermore, ultra-precision lapping is an effective method to obtain high surface quality and accuracy [19]. However, there are only a few studies on the ultra-precision lapping process of the fiber-reinforced PEEK. Therefore, the lapping process based on analyzing the material properties of fiber-reinforced PEEK should be further researched to fully understand the process.

In this paper, the material properties and lapping machinability of fiber-reinforced PEEK has been studied. The effects of the type and mass fraction of modified fibers on the mechanical properties of PEEK have been investigated by analyzing the mechanical properties of PEEK that have been reinforced by carbon fiber and glass fiber. Finally, the lapping experiment was conducted to analyze the influences of property changes of materials on the material removal rate and surface roughness with abrasive paper lapping.

## 2. Materials and Methods

### 2.1. Materials

PEEK and its fiber-reinforced materials were provided by Nanjing Shousu Special Engineering Plastic Products Co., Ltd. (Nanjing, China). In this paper, five kinds of materials were investigated, which include the pure PEEK, carbon-fiber-reinforced PEEK with 10% mass fraction (CF10/PEEK) and 30% mass fraction (CF30/PEEK), glass-fiber-reinforced PEEK with 10% mass fraction (GF10/PEEK) and 30% mass fraction (GF30/PEEK). The average diameter and length of carbon fiber are 8 and 40 μm. The average diameter and length of glass fiber are 10 and 60 μm. The materials were prepared with molding.

### 2.2. Methods

#### 2.2.1. Material Property Tests

Carbon fiber and glass fiber have excellent mechanical properties. The machinability and application of the fiber-reinforced PEEK are influenced by changing the material properties. Therefore, the tensile strength, hardness, and modulus of fiber-reinforced PEEK were tested in this paper to further analyze the influence of material properties on lapping machinability.

The tensile test was conducted at 298K with a tensile rate of 1mm/min using a material testing machine (5500A, Instron, Norwood, MA, USA) to measure the tensile stress curves of the fiber-reinforced PEEK. Flat dog-bone-shaped tensile specimens with a gauge length of 90 mm were made by wire cutting. The tensile specimens were ground on each side with SiC paper, resulting in a final specimen thickness of 4 mm and a gauge section width of 5 mm.

The nanoindenter (TI950, Hysitron, Eden Prairie, MN, USA) with a standard Berkovich indenter was used in this work to measure the load–displacement curves of the fiber-reinforced PEEK materials. The sample was a 15 mm × 15 mm × 5 mm square block made by wire cutting, and the sample surface was polished before the nanoindentation tests. To ensure that the data of the experiment are reliable, three points in a random site of the PEEK matrix on the samples were checked during the test for each indentation depth. The maximum load was 8 mN, with the loading for 10 s, holding for 5 s, and unloading for 10 s. Furthermore, nanoindentation was conducted on the pure PEEK material, carbon fiber, and glass fiber, respectively, with the same conditions.

#### 2.2.2. Lapping Experiment Details

The surface roughness and the material removal rate (MRR) are the most relevant parameters to reflect the lapping machinability of materials. Thus, the MRR and surface roughness with abrasive paper lapping were studied in this work to analyze the influence of fiber types and mass fraction on the lapping machinability of fiber-reinforced PEEK.

**Figure 1:**
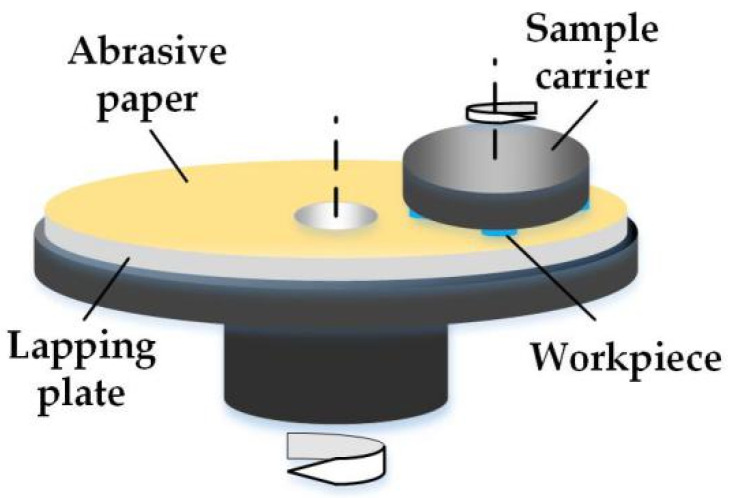
The lapping experimental setup.

All lapping experiments were conducted on an ultra-precision lapping machine (ZYP230, Shenyang Kejing Instrument Inc., Shenyang, China). The sample was a square block made by wire cutting with a size of 15 mm × 15 mm × 10 mm. The different mesh size of silicon carbide abrasive paper was applied in the lapping process to obtain the best surface roughness. During lapping, four samples for the same material were placed on a sample carrier in the once lapping process. A schematic diagram of the lapping experiment is shown in Figure 1.

The lapping parameters for the PEEK machinability analysis are presented in Table 1. The material removal rate (MRR) was calculated by the change of sample thickness per unit time and the sample thickness change during lapping was measured by a laser measuring device (Technological Gocator 2520, LMI, Mississauga, ON, Canada). The surface roughness with abrasive papers of different particle sizes was measured after the lapping process by Talysurf Profiler (CLI2000, Taylor Hobson Ltd., Leicester, UK), and the surface morphology was observed by SEM (SU820, Hitachi, Tokyo, Japan).

## 3. Results

### 3.1. Material Properties of Fiber-Reinforced PEEK

#### 3.1.1. Nanomechanical Properties of Fiber-Reinforced PEEK

**Figure 2:**
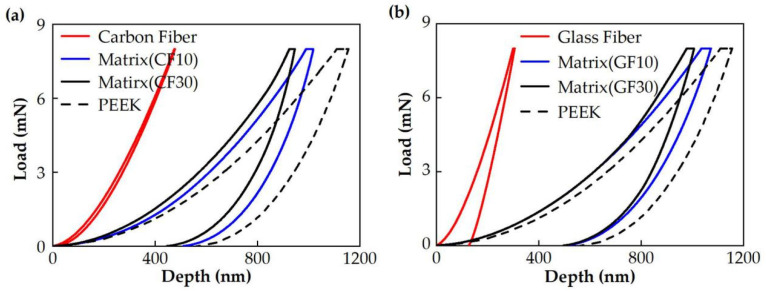
Load–depth (*P*–*h*) curves for (**a**) carbon-fiber-reinforced PEEK (CF/PEEK) materials and (**b**) glass-fiber-reinforced PEEK (GF/PEEK) materials from the nanoindentation tests with a maximum load of 8 mN. The *P*–*h* curves of the pure PEEK, the carbon fiber, and the glass fiber are displayed for comparative analysis.

The components of the fiber-reinforced materials may have a great influence on the mechanical properties. The nanomechanical properties of five kinds of PEEK materials were studied in this work, and their results are exhibited in Figure 2. The nanoindentations in pure PEEK, CF/PEEK, and GF/PEEK show different load–displacement curves.

**Figure 3:**
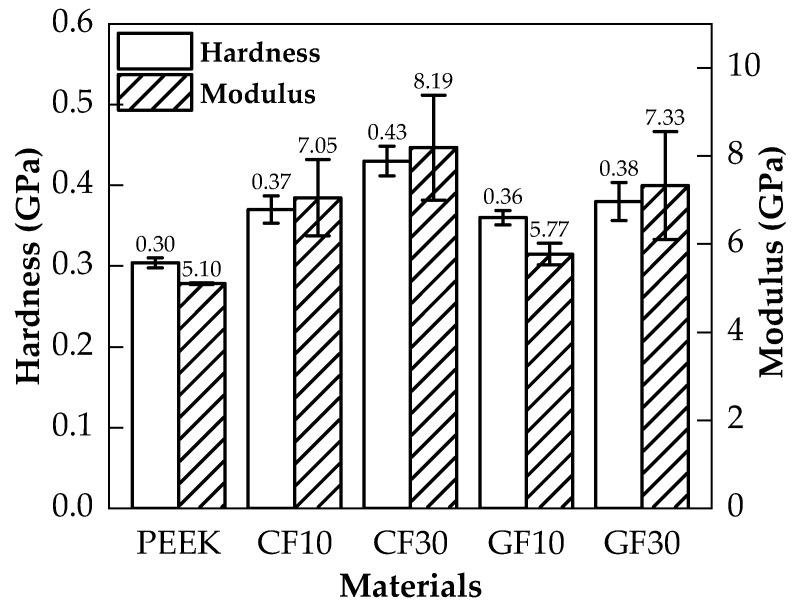
The average hardness and modulus of the pure PEEK and fiber-reinforced PEEK materials.

In Figure 2, the depth of carbon fiber and glass fiber were both smaller than the PEEK with the same load, which proved that the hardness of the fiber was higher than the PEEK. Furthermore, the depth of the fiber-reinforced PEEK matrix was smaller than the pure PEEK, and the influence of the indentation depth was more significant with an increased fiber mass fraction. The hardness and modulus could be calculated based on the Olive-Pharr method [20], and the results are shown in Figure 3. The hardness and modulus of the PEEK matrix and fiber are exhibited in Table 2. As shown in Figure 3 and Table 2, the hardness and modulus of carbon/glass fiber far exceeded that of the PEEK matrix. The hardness and modulus of the PEEK matrix were enhanced by the fiber due to its excellent mechanical properties, and the reinforcement was improved with the increased mass fraction of fiber.

#### 3.1.2. Tensile Mechanical Properties of Fiber-Reinforced PEEK

**Figure 4:**
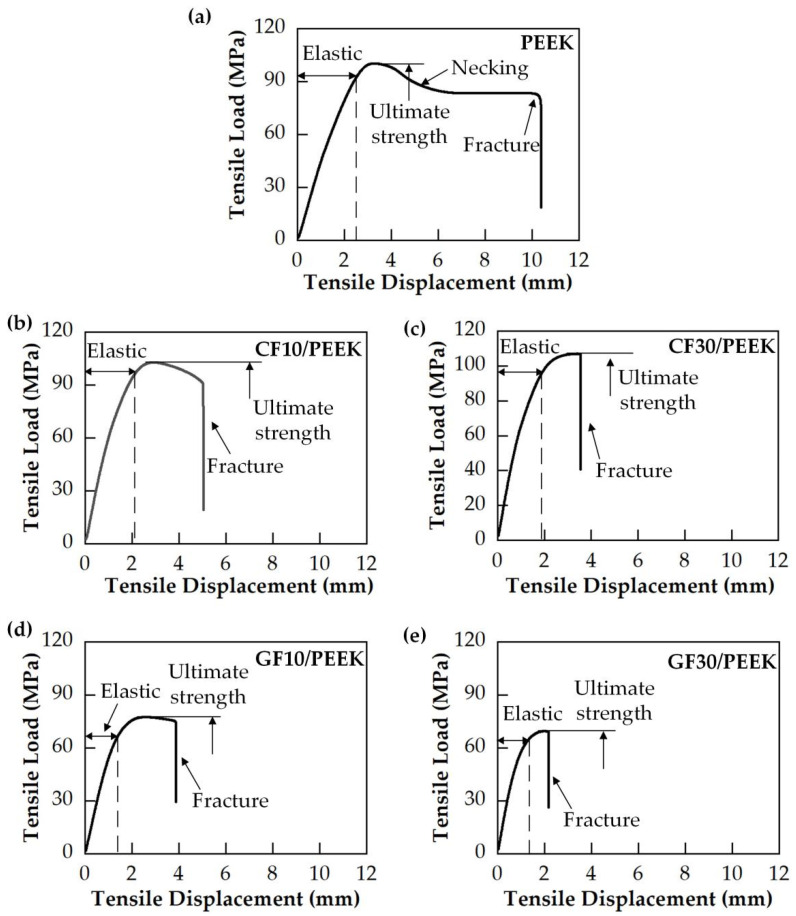
Tensile load–displacement curves of dog-bone-shaped specimens loaded in tension with tensile speeds of 1 mm/min for various materials: (**a**) PEEK; (**b**) carbon-fiber-reinforced PEEK with 10% fiber mass fraction (CF10/PEEK); (**c**) carbon-fiber-reinforced PEEK with 30% fiber mass fraction (CF30/PEEK); (**d**) glass-fiber-reinforced PEEK with 10% fiber mass fraction (GF10/PEEK); (**e**) glass-fiber-reinforced PEEK with 30% fiber mass fraction (GF30/PEEK).

The tensile load–displacement curves of the pure PEEK and fiber-reinforced PEEK materials are shown in Figure 4. The tensile load–displacement curves demonstrated the different deformation phases of fiber-reinforced PEEK materials. In Figure 4, the load of pure PEEK increased with the increase in displacement in the elastic phase. When the load attained the maximum tensile load, the pure PEEK had a necked phenomenon and the load decreased to a constant value until the material failed at the fracture point. The ultimate tensile strength was the value of the maximum tensile load, and the tensile curves proved that the pure PEEK is a ductile material.

The tensile curves of CF/PEEK are represented in Figure 4b. In the elastic phase, the load increased with the displacement. However, necking of the CF/PEEK material occurred, and it fractured immediately when the load attained the ultimate tensile stress. The tensile length of the CF/PEEK fracture was smaller than that of pure PEEK. The tensile length of the material fracture was smaller and the ultimate tensile stress was higher with the increased carbon fiber mass fraction. The tensile curves proved that the CF/PEEK is a brittle material.

Figure 4c demonstrates the tensile curves of GF/PEEK. Similarly, the tensile load increased in the elastic phase until the ultimate tensile stress was attained. The tensile length of the material fracture and ultimate tensile stress became smaller as the glass fiber mass fraction increased. The GF/PEEK is a brittle material. The ultimate tensile stress of five kinds of PEEK materials is exhibited in Table 3. The CF/PEEK had the greatest tensile strength.

### 3.2. Lapping Processing Properties of Fiber-Reinforced PEEK

**Figure 5:**
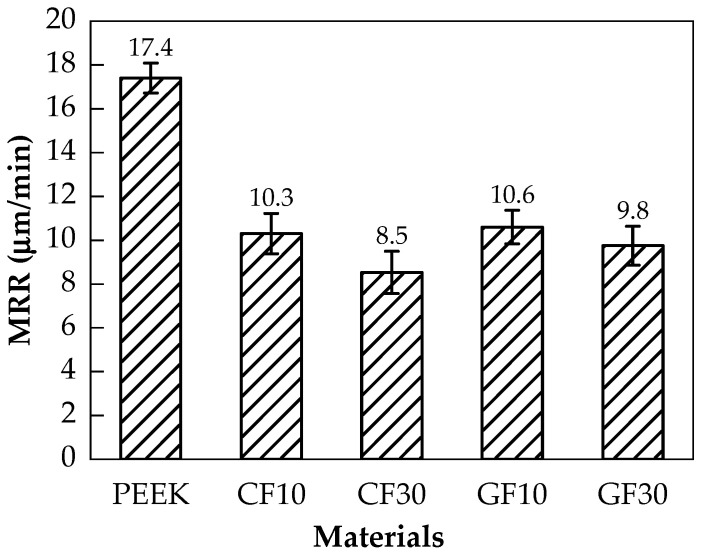
The material removal rates of the pure PEEK and the fiber-reinforced PEEK.

The material removal rate (MRR) reflects the degree of material removal difficulty during the lapping process. Taking the lapping experiment with abrasive paper of #240 mesh size as an example, Figure 5 shows the MRR of pure PEEK and fiber-reinforced PEEK materials. It can be seen in Figure 5 that the maximum MRR was of pure PEEK with the value of 17.4 μm/min. The MRR of CF10/PEEK and CF30/PEEK were 10.3 and 8.5 μm/min, respectively, whereas the MRR of GF10/PEEK and GF30/PEEK were 10.6 and 9.8 μm/min, respectively. As per the data, the MRR during the lapping process showed a decreasing trend when the fiber mass fraction increased.

**Figure 6:**
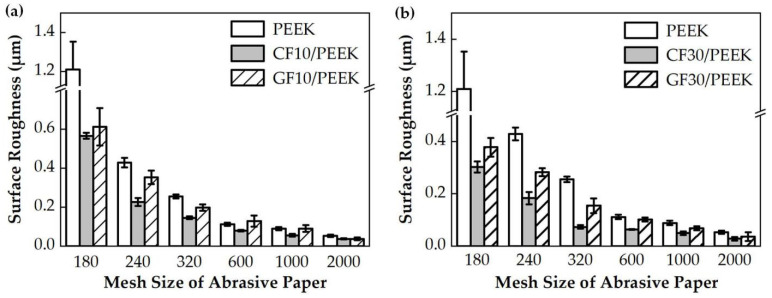
Comparative analysis on surface roughness of fiber types: (**a**) The surface roughness of the pure PEEK, CF10/PEEK, and GF10/PEEK, (**b**) the surface roughness of the pure PEEK, CF30/PEEK, and GF30/PEEK.

**Figure 7:**
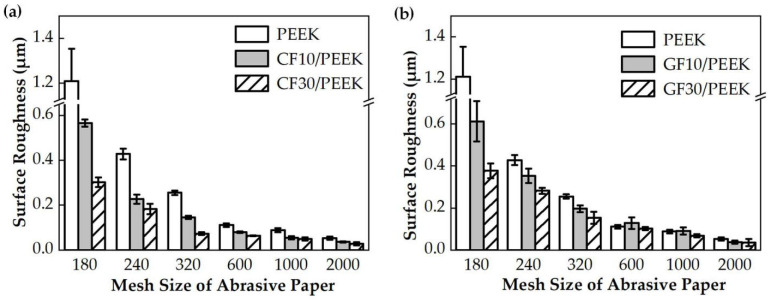
Comparative analysis on surface roughness of the fiber mass fraction: (**a**) The surface roughness of the pure PEEK, CF10/PEEK, and CF30/PEEK, (**b**) the surface roughness of the pure PEEK, GF10/PEEK, and GF30/PEEK.

The surface roughness reflects the quality of the lapping process. The results of surface roughness after the lapping process are shown in Figure 6 and Figure 7. The surface roughness of fiber-reinforced PEEK showed a downward trend with the particle size of the abrasive paper decreasing. When the mesh sizes of abrasive paper were small (#180, #240, #320), the lapped surface roughness was poor, and in addition, the downward trend of the surface roughness was obvious. When the abrasive paper of the #1000 mesh size was applied, the surface roughness improved and the downward trend flattened.

As shown in Figure 6a, with the same mesh size of the abrasive paper, the surface roughness of CF10/PEEK was lower than the GF10/PEEK, which was the same trend between the CF30/PEEK and GF30/PEEK. The CF/PEEK showed better lapping machinability than the GF/PEEK.

Figure 7 compares the surface roughness of different fiber mass fraction reinforced PEEK materials with the same type of fiber. As shown in Figure 7a, the surface roughness of CF30/PEEK was lower than that of CF10/PEEK. Similarly, the surface quality of GF30/PEEK was better than that of GF10/PEEK. The surface quality of fiber-reinforced PEEK improved as the fiber mass fraction increased. As per the data of MRR and surface roughness during the lapping process, the carbon fiber and glass fiber could improve the surface quality but decrease the MRR.

## 4. Discussion

### 4.1. The Influence of Fiber Types on Lapping Machinability of Fiber-Reinforced PEEK Materials

The tensile strength, hardness, and elastic modulus of CF/PEEK materials were larger than the pure PEEK due to the extremely larger hardness and modulus of carbon fiber. The hardness and modulus of glass fiber also far exceeded that of pure PEEK, resulting in improvements in the hardness and modulus of GF/PEEK materials. In contrast, the average length and diameter ratio of glass fibers was too high, which resulted in an easier concentration and formation of defects inside the PEEK matrix and may reduce the tensile strength [21].

**Figure 8:**
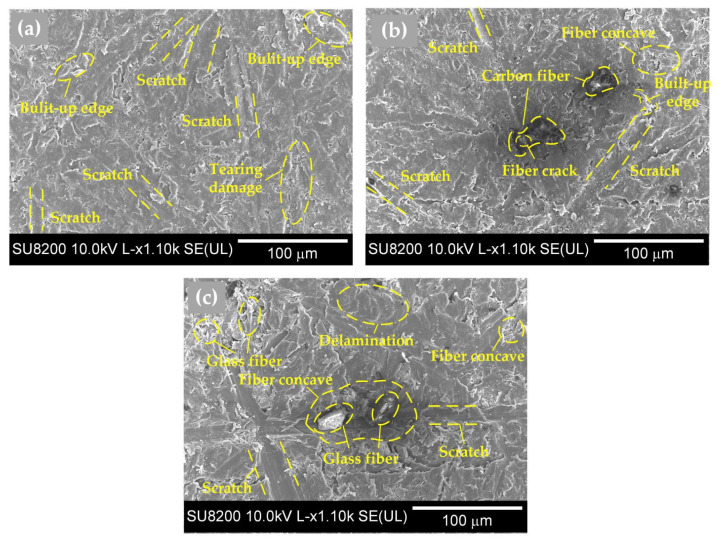
Surface morphology lapped with the #1000 mesh size silicon carbide abrasive paper of various fiber types of reinforced PEEK materials, (**a**) pure PEEK; (**b**) carbon-fiber-reinforced PEEK with the 30% fiber mass fraction; (**c**) glass-fiber-reinforced PEEK with the 30% fiber mass fraction.

As shown in Figure 5, the MRRs of fiber-reinforced PEEK materials were smaller than that of the pure PEEK. During the lapping process, the interaction between abrasive particles and the workpiece could be divided into two categories. The first category was the interaction between abrasive particles and the PEEK matrix, and the second category was the interaction between abrasive particles and reinforcing fibers. The contact between the abrasive particles and the workpiece surface was irregular. Some abrasive particles were in contact with the PEEK matrix, and the others were in contact with the fibers. It can be observed in Table 2 that the hardness of carbon fiber or glass fiber far exceeded that of the pure PEEK. The high hardness of carbon fiber or glass fiber made it more difficult to remove than the pure PEEK during the lapping process, resulting in smaller MRRs of the fiber-reinforced PEEK materials than that of the pure PEEK. In the lapping process, the abrasive particle was coarse and exhibited poor height uniformity. Therefore, the surface roughness significantly decreased, resulting in the quick removal of the material. A rapid reduction in surface roughness would occur when the abrasives were courser. When the abrasive paper with the mesh size of #1000 was applied, the abrasive grains were finer. This improved the high equivalence more, and the pressure of a single abrasive particle on the fiber-reinforced PEEK materials became smaller. The MRR was then reduced, and the surface roughness tended to be better and constant. As shown in Figure 6 and Figure 7, the PEEK had poorer surface quality than the fiber-reinforced PEEK materials with the same mesh size abrasive paper lapping. The lapped surface morphology of CF30/PEEK and GF30/PEEK are shown in Figure 8. The pure PEEK is a ductile material, and the fiber-reinforced PEEK is a brittle material (Figure 4). In Figure 8, there were many built-up edges and delamination on the PEEK matrix surface due to the ductile performance and low strength of the PEEK materials [22]. In the early period of lapping, there was only friction between the workpiece and abrasive particles with no material removal in the workpiece surface, resulting in ductile and elastic deformation. As the abrasive cutting process continued, the PEEK matrix was pressed by the abrasive particle and formed the scratches on the surface [23,24]. It can be observed in Figure 8 that there were many scratches and built-up edges on the lapped surface.

In Figure 8b, the scratch also occurred on the carbon fiber surface, but the scratch depth was shallower than the pure PEEK due to its high hardness. During the lapping process, the material removal methods of carbon fiber included cracks, scratches, and desquamations from the matrix [25]. The concaves formed by fiber desquamation were covered by the PEEK matrix as the lapping process continued; therefore, the fiber-reinforced PEEK had better surface quality than the pure PEEK with the same mesh size abrasive paper lapping.

In Figure 6, the CF/PEEK attained better surface quality than the GF/PEEK with the same fiber mass fraction. On the one hand, in Table 2, the hardness of carbon fiber was higher than the glass fiber. With the same mesh size abrasive paper lapping, the carbon fiber was more difficult to remove than the glass fiber, and the scratches on the carbon fiber were shallower than the glass fiber. Moreover, the average diameter and length of glass fiber were larger than that of carbon fiber, and in Figure 8c, the carbon fiber concaves formed by fiber desquamation were easier to cover by the PEEK matrix, resulting in better surface quality and a smaller MRR.

On the other hand, the reinforcement of fiber on the PEEK matrix varied with the fiber types. In Figure 3, the hardness and modulus of CF30/PEEK were higher than that of GF30/PEEK. Therefore, deformation of CF30/PEEK was more difficult, and the scratches of CF/PEEK would be shallower than the GF/PEEK. The CF/PEEK can attain better surface quality, whereas the CF/PEEK matrix attained higher hardness, which made it more difficult to be removed than the glass fiber, the MRR of CF/PEEK was smaller than that of GF/PEEK.

### 4.2. The Influence of Fiber Mass Fraction on Lapping Machinability of Fiber-Reinforced PEEK Materials

In Figure 2 and Figure 3, the hardness and modulus of the PEEK matrix improved significantly as the fiber mass fraction increased. The more carbon fiber or glass fiber mass fraction, the more the fiber was widely distributed on the PEEK matrix surface, which improved the hardness and modulus of fiber-reinforced PEEK materials. In Figure 4, the fiber-reinforced PEEK materials performed as brittle materials due to the brittleness of carbon and glass fiber. The tensile length of the fracture point decreased with the fiber mass fraction. The carbon fiber could improve the ultimate tensile strength of CF/PEEK. However, the length and diameter ratio of glass fiber was too large to form the defects inside the PEEK, resulting in the decrease in the ultimate tensile strength. Furthermore, the ultimate tensile strength decreased as the fiber mass fraction increased.

In Figure 5 and Figure 7, the fiber-reinforced PEEK materials that had more fiber mass fraction could attain a smaller MRR and a better surface quality. In terms of MRRs of the same fiber type reinforced with PEEK of different fiber mass fractions, the more fiber mass fraction, the wider the interaction area between the abrasive particles and the fiber would be. Furthermore, the hardness of the PEEK matrix would be improved with an increased fiber mass fraction, resulting in the decreasing trend of the MRR due to the high hardness of the fiber.

**Figure 9:**
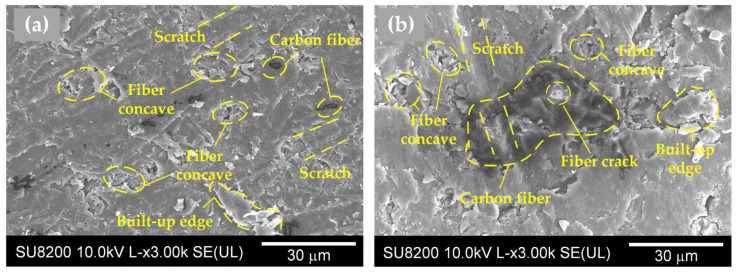
Surface morphology lapped with the #1000 mesh size silicon carbide abrasive paper of carbon-fiber-reinforced PEEK materials with different carbon fiber mass fractions, (**a**) carbon-fiber-reinforced PEEK with 10% carbon fiber mass fraction; (**b**) carbon-fiber-reinforced PEEK with 30% carbon fiber mass fraction.

Figure 9 shows the surface morphology of different carbon fiber mass fraction reinforced PEEK materials. As shown in Figure 2a, the nanoindentation curves of CF30/PEEK were above the CF10/PEEK, demonstrating that CF30/PEEK has a better ability to resist deformation. The scratches on the CF30/PEEK matrix were shallower than the CF10/PEEK. On the other hand, the carbon fiber could attain better surface quality than the PEEK matrix. Therefore, the improvement effect on the surface quality was more significant with an increased carbon fiber mass fraction.

**Figure 10:**
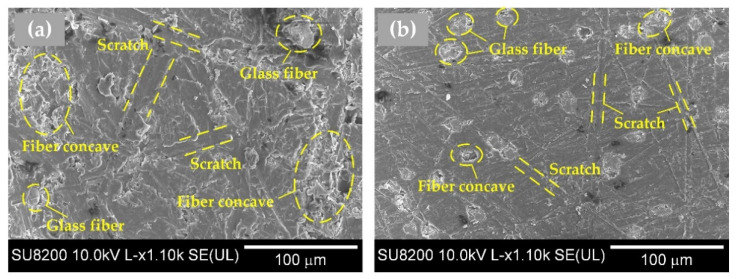
Surface morphology lapped with the #1000 mesh size silicon carbide abrasive paper of glass-fiber-reinforced PEEK materials with different glass fiber mass fractions, (**a**) glass-fiber-reinforced PEEK with 10% glass fiber mass fraction; (**b**) glass-fiber-reinforced PEEK with 30% glass fiber mass fraction.

The lapped surface morphology of GF10/PEEK and GF30/PEEK are shown in Figure 10. In Figure 2b and Figure 3, the GF30/PEEK had superior mechanical properties to the GF10/PEEK. In Figure 10a, the scratches on the GF10/PEEK matrix were wider and more densely distributed, which led to poor surface quality, and there was tearing damage and material accumulation near the glass fiber concaves due to the ductile performance of the PEEK matrix. In Figure 10b, the glass fiber was distributed wider and the mechanical properties of PEEK matrix were improved significantly more than the CF10/PEEK. The scratches became shallow and narrow. The tearing damage phenomenon was greatly weakened and the surface quality improved.

## 5. Conclusions

In this paper, tensile tests, nanoindentation tests, and lapping experiments were carried out on five varieties of PEEK and its fiber-reinforced materials. The influences of the types and mass fraction of the modified fibers on the material properties were investigated in this work, including tensile strength, hardness, modulus, and lapping machinability. The following conclusions have been drawn from the results.

(1) In terms of material properties for CF/PEEK, carbon fiber has high hardness and modulus and is evenly distributed inside the material. Thus, the tensile strength, hardness, and modulus are improved. For GF/PEEK, the glass fiber is easy to concentrate and form defects inside the material due to its high hardness, modulus, and brittleness. This, in turn, decreases the tensile strength and improves the hardness and modulus. The influences on tensile strength and nanomechanical properties are more significant as the fiber mass fraction increases.
(2) In the lapping process, fiber-reinforced PEEK has better surface quality, and the MRR is lower than the pure PEEK due to the superior mechanical properties of the fiber. The lapped surface quality of CF/PEEK is better than GF/PEEK. Because the carbon fiber has higher hardness and modulus than the glass fiber, this results in a weaker deformation on the PEEK matrix surface.
(3) The higher the mass fraction is, the more the mechanical properties of fiber-reinforced PEEK are improved. As the fiber reinforcing can improve the lapped surface quality and hardness of the PEEK matrix, the fiber-reinforced PEEK with a higher fiber mass fraction has a better lapped surface quality and lower MRR.

## Figures and Tables

**Table 1:** Lapping parameters for fiber-reinforced PEEK lapping machinability analysis.

Lapping Condition	Value
Abrasive	Silicon carbide abrasive paper
Mesh size	180, 240, 320, 600, 1000, 2000
Lapping plate speed (r/min)	40
Workpiece speed (r/min)	63
Lapping time (min)	40
Lapping pressure (KPa)	11

**Table 2:** The average hardness and modulus of the pure PEEK and carbon/glass fiber.

Sample	PEEK	Carbon Fiber	Glass Fiber
Hardness (GPa)	0.3	3.3	2.9
Modulus (GPa)	5.06	16.52	26.71

**Table 3:** The ultimate tensile strength of the pure PEEK and fiber-reinforced PEEK materials.

Materials	PEEK	CF10/PEEK	CF30/PEEK	GF10/PEEK	GF30/PEEK
Ultimate tensile strength (MPa)	98.1	99.5	102.6	78.6	61.0

## Data Availability

The data presented in this study are available on request from the corresponding author.
